# Electrospun Polycaprolactone/Gelatin Blended Nanofibre Textiles with Controlled Dexamethasone Release for Anti-Inflammatory Wound Dressings

**DOI:** 10.3390/polym18121495

**Published:** 2026-06-14

**Authors:** Md Raihan Hossain, Mohammad Mahbubul Alam, Carola Esposito Corcione, Raffaella Striani, Md. Shamim Alam

**Affiliations:** 1 Department of Macromolecule Chemistry & Physics, Northeast Normal University, Renmin Street 5268, Changchun 130024, China; 2 Department of Textile Engineering, Ahsanullah University of Science and Technology, Dhaka 1208, Bangladesh; mahbub_tex@aust.edu; 3 Department of Innovation Engineering, University of Salento, 73100 Lecce, Italy; carola.corcione@unisalento.it; 4 Department of Textile Engineering, Southeast University, Tejgaon, Dhaka 1208, Bangladesh

**Keywords:** electrospinning, polycaprolactone/gelatin, surface crystallization, anti-inflammatory, wound dressing

## Abstract

The performance of drug-loaded electrospun nanofibres is governed not only by drug content but also by the spatial distribution of the drug within the fibre matrix, which determines release kinetics and biological response. Here, we demonstrate that dose-dependent surface crystallisation of dexamethasone (DEX) in electrospun polycaprolactone (PCL)/gelatin nanofibres controls drug release behaviour and subsequent macrophage-mediated inflammation. Nanofibre mats containing 0, 1, and 2 wt% DEX (PG0, PG1, PG2) were fabricated and systematically characterised. Scanning electron microscopy revealed a change from homogeneous fibres (PG0) to surface-decorated crystalline domains with increasing drug loading, which indicates a supersaturation-driven phase separation during electrospinning. This morphological evolution directly governs the transport behaviour: PG2 exhibits a pronounced burst release due to surface-localised drug, whereas PG1 shows a balanced release profile with both surface-accessible and matrix-embedded drug fractions. Release characteristics result in different biological outcomes. PG1 and PG2 strongly inhibit pro-inflammatory cytokines (TNF-α and IL-6) in LPS-stimulated macrophages (~70–75% reduction), confirming retained drug bioactivity. However, higher drug loading (PG2) leads to lower fibroblast viability and compromised mechanical integrity. Importantly, PG1 shows a desirable balance of controlled drug release, cytocompatibility (>90% viability) and mechanical performance (~8 MPa) with effective anti-inflammatory activity. Degradation studies also show controlled structural evolution without destabilisation upon pH change, demonstrating suitability for wound environments. These results reveal surface crystallisation as an important design parameter for electrospun drug delivery systems and demonstrate that optimal therapeutic performance is controlled by intermediate drug loading, not maximum loading, providing a mechanistic framework for the rational design of immunomodulatory wound dressings.

## 1. Introduction

Chronic wounds, such as diabetic ulcers and pressure injuries, exhibit persistent inflammation, which prevents the normal healing cascade and severely impairs tissue regeneration. Pro-inflammatory macrophages that are persistently activated produce cytokines such as tumour necrosis factor-α (TNF-α) and interleukin-6 (IL-6), which prevents the transition to the proliferative phase of healing [1,2,3]. While systemic administration of anti-inflammatory agents can suppress this response, it is often associated with off-target effects and insufficient drug localisation at the wound site [4,5]. These limitations have driven the development of localised drug delivery platforms capable of modulating the wound microenvironment in a controlled and sustained manner.

Electrospun nanofibre scaffolds are attractive for such application because of their high surface area, interconnected porosity and structural similarity to the extracellular matrix (ECM) that promote cell interaction and efficient drug delivery [6,7,8]. Polycaprolactone (PCL) has been selected among the available material systems due to its mechanical stability and slow degradation; gelatin has been chosen to improve hydrophilicity and provide bioactive motifs for cell adhesion and proliferation [9,10,11]. Therefore, PCL/gelatin hybrid nanofibres have been widely studied for wound-healing applications. However, current studies tend to focus on drug loading and the overall release characteristics, with less emphasis on the influence of drug distribution within the fibre matrix on the release characteristics and biological response.

Electrospinning is one of the most versatile techniques for producing ultrafine fibres with diameters ranging from the nanometre to micrometre scale. The resulting fibrous structures possess high specific surface area, interconnected porosity, and morphological similarity to the extracellular matrix, making them particularly attractive for wound healing, tissue engineering, and drug delivery applications. Numerous polymeric systems have been electrospun for controlled therapeutic delivery; however, understanding how drug distribution within electrospun fibres influences biological performance remains an ongoing challenge.

An important but less-studied aspect of electrospun drug delivery systems is the phase behaviour of the drug during fibre formation. Rapid evaporation of the solvent may cause supersaturation, leading to phase separation and crystallisation of the drug inside or on the surface of the fibres [12,13,14]. This heterogeneous distribution has a great impact on drug accessibility and often leads to biphasic release profiles, where an initial burst release of surface-associated drug is followed by sustained diffusion from the polymer matrix [15,16]. However, the direct relationship between drug phase distribution, release kinetics, and immunomodulatory function is not well understood while these observations are made.

Dexamethasone (DEX) is a potent glucocorticoid and is widely used to suppress inflammatory signalling pathways and cytokine production in activated macrophages [17,18,19]. Electrospun DEX-loaded scaffolds have been promising in reducing inflammation and improving healing outcomes [20,21]. However, an increase in drug loading does not necessarily translate into an improved therapeutic performance, as excessive drug concentrations can lead to cytotoxic effects or compromise the scaffold integrity [22,23]. This indicates a dose-dependent window of performance with drug loading to be optimised rather than maximised.

Although PCL/gelatin electrospun systems containing dexamethasone have been previously reported for tissue engineering and wound-healing applications, most studies have primarily focused on drug loading efficiency, release duration, and therapeutic outcomes. Comparatively little attention has been given to understanding how drug distribution within the fibre matrix evolves with increasing drug content and how this distribution affects release mechanisms and biological performance. In particular, the influence of dose-dependent surface crystallisation on drug accessibility, macrophage modulation, and cytocompatibility remains insufficiently understood. Addressing this knowledge gap is essential for establishing rational design criteria for electrospun anti-inflammatory drug delivery systems.

Here, we show that the surface crystallisation of dexamethasone in electrospun PCL/gelatin nanofibres is dose-dependent and controls the drug release kinetics and the macrophage-mediated inflammatory response. We systematically vary DEX content (0, 1 and 2 wt%) to establish a direct correlation between fibre morphology, release mechanism and biological outcome. We demonstrate that intermediate drug loading leads to a balanced system with controlled release, high cytocompatibility and efficient anti-inflammatory activity, while high loading results in excessive burst release and decreased cell viability.

These results set a mechanistic basis for the rational design of electrospun drug delivery systems and show that surface crystallisation rather than drug content is the key determinant of therapeutic performance.

The objective of this study was to investigate the influence of dexamethasone loading on fibre morphology, drug phase distribution, release behaviour, mechanical performance, cytocompatibility, and anti-inflammatory activity in electrospun PCL/gelatin nanofibres. Particular attention was given to understanding how drug distribution within the fibre matrix affects therapeutic performance.

## 2. Experimental Methodology

### 2.1. Materials

Polycaprolactone (PCL, Mn ≈ 80 kDa), gelatin (Type A, porcine skin, bloom ~300), and dexamethasone (DEX, ≥98%) were obtained from Sigma-Aldrich (St. Louis, MO, USA).

1,1,1,3,3,3-Hexafluoro-2-propanol (HFIP, ≥99%) was used as the solvent.

Lipopolysaccharide (LPS, *E. coli* O111:B4), MTT reagent, calcein-AM, propidium iodide (PI), Hoechst 33342, paraformaldehyde, Triton X-100, and bovine serum albumin (BSA) were also sourced from Sigma-Aldrich.

Dulbecco’s Modified Eagle Medium (DMEM), foetal bovine serum (FBS), penicillin–streptomycin, and trypsin–EDTA were supplied by Gibco (Grand Island, NY, USA). Mouse TNF-α and IL-6 ELISA kits were obtained from R&D Systems (Minneapolis, MN, USA). All chemicals were used as received without any further purification.

### 2.2. Preparation of Electrospun Nanofibres

The polymer solution was prepared by dissolving PCL and gelatin in HFIP at a weight ratio of 70:30 to achieve a total polymer concentration of 12 wt%. The PCL/gelatin ratio of 70:30 was selected based on previous reports demonstrating an effective balance between the mechanical strength and structural stability provided by PCL and the hydrophilicity and cell-interactive properties contributed by gelatin. This composition has also been reported to provide stable electrospinning behaviour and suitable fibre formation for biomedical applications. The solution was stirred at room temperature for 12 h until homogeneous. Dexamethasone was then added at 0, 1 and 2 wt% relative to polymer content to obtain formulations PG0, PG1 and PG2, respectively.

Electrospinning was performed using a syringe pump with a 21-gauge stainless steel needle. The solution was infused at a rate of 0.5 mL h-1 at 15 kV and the distance between the tip and collector was 15 cm. Fibres were collected on a rotating drum at 500 rpm in controlled conditions (25 °C, 40 ± 5% RH).

**Figure 1:**
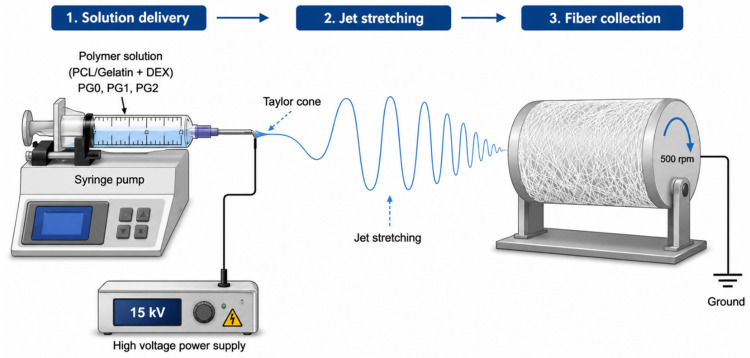
Schematic illustration of the electrospinning process used for fabrication of PCL/gelatin/dexamethasone nanofibres, showing solution delivery, jet stretching under an applied electric field, and fibre collection on a rotating drum collector.

The collected nanofibre mats were vacuum-dried for 48 h to remove residual solvent and stored in a desiccator prior to use. CL/gelatin/dexamethasone nanofibres were fabricated using an electrospinning system, as schematically illustrated in Figure 1.

### 2.3. Fourier Transform Infrared (FTIR) Analysis

FTIR spectroscopy (Nicolet Nexus 670, Thermo Fisher Scientific, Waltham, MA, USA) was performed to analyse the chemical structure of the materials and to evaluate potential interactions within the electrospun nanofibres. Spectra of PCL, gelatin, and electrospun samples (PG0, PG1, and PG2) were recorded using an FTIR spectrometer in the range of 4000–500 cm^−1^ at a resolution of 4 cm^−1^ with 32 scans per sample.

All samples were analysed using attenuated total reflectance (ATR) mode without further preparation. Background correction was performed prior to each measurement, and the spectra were normalised for comparison.

### 2.4. Morphological Characterisation

Fibre morphology was analysed using field-emission scanning electron microscopy (FE-SEM) (ZEISS Gemini VD350 SEM, ZEISS, Oberkochen, Germany). Samples were sputter-coated with a thin layer (~15 nm) of gold–palladium prior to imaging.

Fibre diameter was quantified using ImageJ software (Version 1.54, National Institutes of Health, Bethesda, MD, USA) by measuring at least 50 fibres per sample from randomly selected regions. Results are reported as mean ± standard deviation.

### 2.5. Mechanical Testing

Mechanical properties were measured using a universal testing machine (Instron 3342, Instron Corporation, Norwood, MA, USA) equipped with a 10 N load cell. The thickness was measured at five points on rectangular specimens of 50 mm × 10 mm.

The tensile tests were carried out at a crosshead speed of 10 mm min^−1^ with an initial gauge length of 30 mm. Each formulation was tested on at least five samples (*n* = 5). Young’s modulus, tensile strength and elongation at break were calculated from stress–strain curves.

### 2.6. In Vitro Drug Release Study

Nanofibre mats (20 mg) were immersed in 10 mL of phosphate-buffered saline (PBS, pH 7.4) and incubated at 37 °C with gentle shaking (50 rpm). At pre-determined time points, 1 mL aliquots were removed and replaced with an equal volume (1 mL) of fresh PBS to maintain sink conditions.

The concentration of DEX was determined by UV-Vis spectroscopy at 242 nm using a calibration curve. The cumulative release was expressed as a percentage of the total drug content. A calibration curve for dexamethasone was established at 242 nm within the concentration range used for release analysis. The release measurements were performed under identical experimental conditions for all formulations to enable comparative evaluation. Full analytical validation, including LOD, LOQ, specificity, and interference studies, was beyond the scope of the present work and will be considered in future investigations.

Solution rheological and electrohydrodynamic parameters, including viscosity, conductivity, and surface tension, were not quantitatively measured in this study. Although stable fibre formation was achieved for all formulations, future work should investigate these parameters to establish direct relationships between solution properties, phase behaviour, and fibre morphology.

Release kinetics was studied with the normalised release fraction (Mt/M∞).

### 2.7. Cell Culture

L929 mouse fibroblasts and RAW 264.7 macrophages were grown in DMEM with 10% FBS and 1% penicillin–streptomycin at 37 °C in a humidified atmosphere of 5% CO_2_.

Passage numbers 5–12 were used for the cells. Cell seeding density was normalised to scaffold surface area to ensure consistent cell–material interactions.

### 2.8. Cytocompatibility (MTT Assay)

Prior to cytocompatibility testing, the electrospun nanofibre mats were sterilised by exposure to ultraviolet (UV) light for 30 min on each side under aseptic conditions. The sterilised samples were subsequently used for extract preparation and cell culture experiments.

The cytocompatibility was evaluated using the indirect extract method according to the ISO 10993-5 standard [24]. To obtain extracts, nanofibre mats (0.01 g mL^−1^) were incubated in culture medium for 24 h.

L929 cells were seeded at 1 × 10^4^ cells/well and treated with the extracts for 24, 48, and 72 h. MTT reagent was added and incubated for 4 h. The formazan crystals were dissolved in DMSO. The absorbance was read at 570 nm.

Cell viability was calculated as compared to untreated controls.

### 2.9. Live/Dead Staining

Cells cultured on nanofibre mats for 48 h were stained with calcein-AM (2 μM) and propidium iodide (4 μM) for 30 min at 37 °C. Samples were imaged using fluorescence microscopy to assess live (green) and dead (red) cells.

### 2.10. Cell Adhesion and Morphology

The cells cultured for 24 h were fixed in 4% paraformaldehyde, permeabilised with Triton X-100, and blocked in BSA. F-actin was stained with phalloidin, and nuclei were stained with Hoechst 33342.

Fluorescence microscopy (BX53, Olympus Corporation, Tokyo, Japan) was used to evaluate cell spreading and cytoskeletal organisation.

### 2.11. Anti-Inflammatory Assay

RAW 264.7 macrophages were seeded on nanofibre mats and stimulated with LPS (1 μg mL^−1^) for 48 h. Supernatants were collected and analysed for TNF-α and IL-6 using ELISA kits according to the manufacturer’s instructions.

### 2.12. Degradation Study

Nanofibre mats (20 mg) were incubated in PBS (pH 7.4) at 37 °C. PBS was replaced every 48 h to maintain the pH constant and to prevent the accumulation of degradation byproducts. Samples were collected at 0, 7, 14 and 21 days, washed, lyophilised and weighed.

Mass loss (%) was calculated as a function of initial weight. The pH of the degradation medium was controlled during the study.

The fibre integrity was scored semi-qualitatively from SEM images taken at each time point according to the following criteria: 4 = intact fibres with smooth surface; 3 = minor surface erosion; 2 = partial fibre swelling and fusion; 1 = extensive fragmentation; and 0 = complete loss of fibrous structure. Scoring was performed by three independent blinded evaluators, and the mean score is reported.

### 2.13. Statistical Analysis

Data are expressed as mean ± standard deviation (SD) (*n* ≥ 3). Statistical significance was determined using one-way ANOVA with Tukey’s post hoc test. Differences were considered significant at *p* < 0.05.

For datasets involving both formulation and time as independent variables, future studies may employ two-way ANOVA to evaluate interaction effects and provide a more comprehensive statistical interpretation.

## 3. Results and Discussion

### 3.1. Electrospinning Stability and Fibre Formation Window

All the formulations in the electrospinning process led to continuous and defect-free nanofibres (Figure 1), indicating that the selected parameter space (voltage, flow rate and tip-to-collector distance) is in a stable electrohydrodynamic regime. Incorporation of up to 2 wt% dexamethasone (DEX) does not induce jet breakup or bead formation, indicating that the solution possesses enough chain entanglement and viscoelasticity for fibre formation.

This stability results from the complementary roles of the polymer components: PCL provides the long-chain entanglement for jet continuity, and gelatin increases the solution conductivity to guarantee uniform charge distribution along the jet. The absence of any morphological defects in PG0-PG2 indicates that the incorporation of drug does not affect the jet dynamics, but it affects the phase behaviour after solidification, as will be seen in later morphological analysis.

### 3.2. Chemical Structure and Phase Distribution (FTIR Analysis)

**Figure 2:**
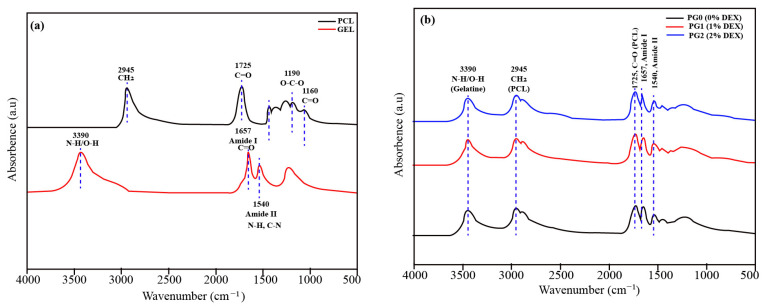
FTIR analysis of pristine components and electrospun nanofibres. (**a**) FTIR spectra of PCL, gelatin, and blended PCL/gelatin nanofibres (PG0). (**b**) FTIR spectra of DEX-loaded nanofibres (PG0, PG1, and PG2).

FTIR spectra of the as-received components and the electrospun nanofibres are shown in Figure 2a,b, respectively. As shown in Figure 2a, PCL shows characteristic CH2 stretching bands at ~2940 and ~2865 cm^−1^ and a strong ester carbonyl peak at ~1725 cm^−1^; gelatin shows a broad O–H/N–H band centred at ~3440 cm^−1^ and amide I (~1650 cm^−1^) and amide II (~1540 cm^−1^) bands. The chemical structures are preserved during the electrospinning, as evidenced by the existence of these features in the mixed nanofibres (PG0). A slight broadening in the hydrogen bonding region indicates weak intermolecular interactions between PCL and gelatin, consistent with formation of a physically integrated hybrid matrix.

The spectra of DEX-loaded fibres (Figure 2b) do not show any new vibrational bands or significant shifts in peaks with increasing drug content, which suggests that DEX is incorporated without chemical alteration of the polymer network. Weak secondary interactions between DEX and the surrounding matrix are indicated by minor variations in peak intensity, especially in the 3200–3500 cm^−1^ and 1650–1730 cm^−1^ regions.

**Figure 3:**
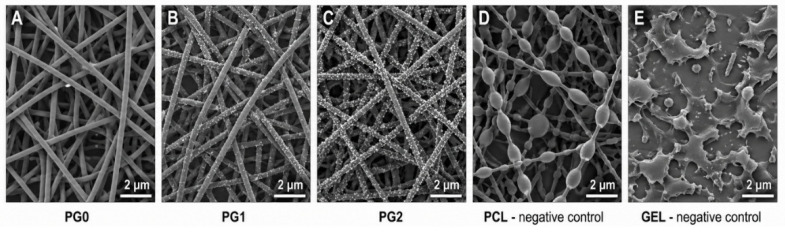
Morphological characterisation of electrospun nanofibres. (**A**) PG0 (0 wt% DEX), (**B**) PG1 (1 wt% DEX), (**C**) PG2 (2 wt% DEX), (**D**) PCL, and (**E**) Gelatin. Scale bar = 2 μm.

Importantly, the lack of significant spectral shifts means a poor miscibility of DEX at the molecular level in the PCL/gelatin system. This behaviour suggests that drug incorporation is mainly due to physical encapsulation rather than molecular dispersion. When combined with the morphological observations, this limited miscibility (Figure 3A–C) favours the formation of heterogeneous drug domains, particularly at higher loading, where segregation into surface-associated regions becomes energetically favourable [25].

This structural arrangement offers a mechanistic basis for the observed release behaviour. The weakly interacting, spatially segregated drug domains are consistent with the evolution from smooth fibres (Figure 3A, PG0) to surface-decorated fibres (Figure 3C, PG2), and enable the dual transport pathways, where the surface-accessible DEX contributes to the initial release, whereas the sustained diffusion is dictated by the matrix-embedded fractions. Thus, FTIR analysis combined with morphological evidence suggests that drug–polymer interactions are not the dominant factor controlling release; rather, phase distribution controls the transport mechanism [26].

Overall, the FTIR data confirm the physical embedding of DEX in the PCL/gelatin matrix without chemical modification and weak drug–polymer interactions, which lead to the phase separation behaviour shown in Figure 3.

### 3.3. Dose-Dependent Surface Crystallisation and Phase Distribution

SEM images are shown in Figure 3A–C, showing the gradual transformation of the homogeneous fibres into phase-separated and surface-decorated structures with increasing DEX loading. The PG0 has smooth and homogeneous morphology, which suggest that PG0 is a single-phase polymer matrix. In contrast, PG1 shows isolated nanoscale protrusions and PG2 shows a dense distribution of surface-associated particulate domains morphologically consistent with drug crystallisation on the fibre surface.

Crucially, statistically, no change in fibre diameter occurs in all samples (*p* > 0.05), implying that the electrospinning dynamics are decoupled from drug-induced morphological change. Instead, the observed features are due to phase separation caused by supersaturation during rapid evaporation of the solvent, when the limited solubility of the drug in the polymer matrix induces nucleation and crystallisation [27]. The absence of statistically significant diameter differences suggests that the relatively low dexamethasone concentrations employed did not substantially alter the electrohydrodynamic properties governing jet stretching during electrospinning. Consequently, the observed surface features are attributed to drug phase separation phenomena rather than changes in overall fibre dimensions.

At lower loading (PG1), drug domains are still partially embedded, while at higher loading (PG2), the system crosses the critical supersaturation threshold, promoting preferential migration of DEX towards the fibre–air interface. This leads to surface-localised crystalline reservoirs, fundamentally changing transport behaviour.

Control samples (Figure 3D,E) highlight the necessity of the hybrid system. Pure PCL shows bead formation because of its low conductivity and gelatin lacks mechanical integrity. These data confirm that only the PCL/gelatin combination enables stable fibre formation and controlled phase behaviour.

### 3.4. Mechanical Integrity and Microstructural Disruption

**Figure 4:**
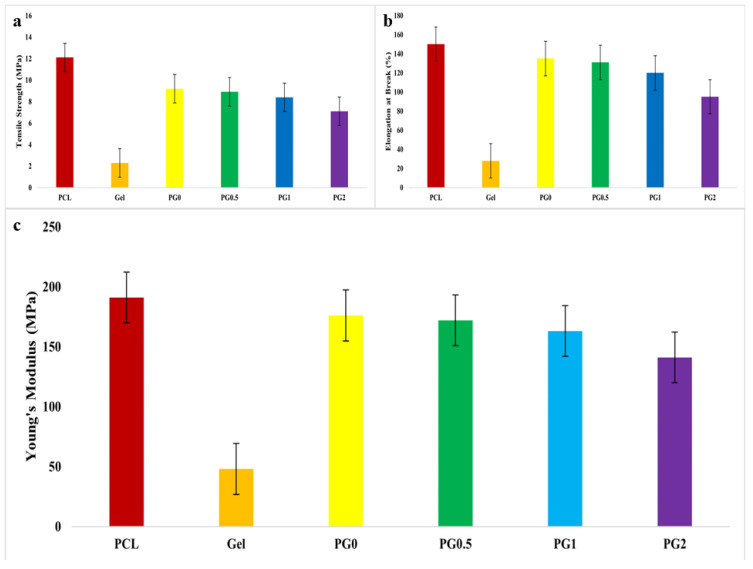
Mechanical properties of electrospun nanofibre mats. (**a**) Tensile strength, (**b**) elongation at break, and (**c**) Young’s modulus for PG0, PG1, and PG2 formulations.

The mechanical characterisation (Figure 4a–c) shows a monotonic decrease in the tensile strength, elongation and modulus with the increment of DEX content. PG0 has the best mechanical properties, while PG1 and PG2 have decreasing values corresponding to increasing structural heterogeneity. The balanced mechanical behaviour observed in the PCL/gelatin nanofibres originates from the complementary contributions of both polymers. PCL provides flexibility and deformation capability, whereas gelatin contributes stiffness through intermolecular interactions within the hybrid network. As a result, the material exhibits both a relatively high modulus and appreciable elongation at break compared with purely brittle systems. Two coupled mechanisms control this behaviour:

➢ Disruption at the interface: DEX domains disrupt the intermolecular interactions between PCL and gelatin, reducing cohesive strength and load transfer efficiency.
➢ Defect-mediated failure: Surface crystals serve as stress concentrators inducing localised fracture under tensile loading.

All formulations, however, still show tensile strength above the functional threshold for wound dressing applications, indicating that mechanical performance is still application-relevant at higher drug loading. It is remarkable that PG1 shows a good balance between structural integrity and functional modification, whereas PG2 is close to the limit where the defect density starts to control the mechanical response [28].

### 3.5. Cytocompatibility and Dose-Dependent Cellular Response

**Figure 5:**
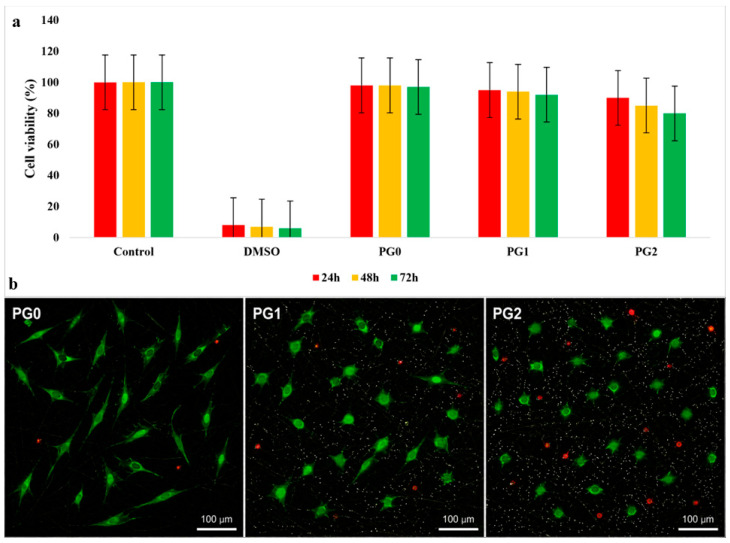
Evaluation of cytocompatibility of nanofibre extracts and surfaces. (**a**) MTT assay for L929 fibroblast cell viability at 24, 48 and 72 h. (**b**) Live/dead fluorescent images after 48 h (green: live cells; red: dead cells). Scale bar = 100 μm (for all the panels in Figure 5b).

The cytocompatibility results (Figure 5a) demonstrate that PG0 and PG1 preserve high cell viability (>90%) for 72 h, while PG2 shows a moderate but significantly decreased (~75%) cell viability. These results are confirmed by live/dead imaging (Figure 5b), with more dead cells at higher DEX concentration.

This trend is analogous to the well-characterised dose-dependent biological response to glucocorticoids, in which low concentrations induce anti-inflammatory effects without impairing cell proliferation, whereas higher concentrations can result in cytotoxicity. It is important to mention that all samples are above the ISO cytotoxicity threshold, confirming an overall biocompatibility [29].

These findings point to a critical trade-off: higher drug loading improves therapeutic efficacy but narrows the cytocompatibility window, implying that optimised dosing is preferred over maximal loading.

PG1 maintains a high level of cytocompatibility similar to control, while PG2 shows a moderate decrease owing to higher drug loading. Data are represented as mean ± SD (*n* ≥ 3).

### 3.6. Cell Adhesion and Cytoskeletal Adaptation

Fluorescence images of fibroblasts attaching and spreading well on PG0 and PG1 nanofibres show well-defined actin networks and organised nuclei. The mild rounding with respect to planar controls indicates an adaptation to the three-dimensional fibrous architecture, providing topographical cues rather than flat substrates.

**Figure 6:**
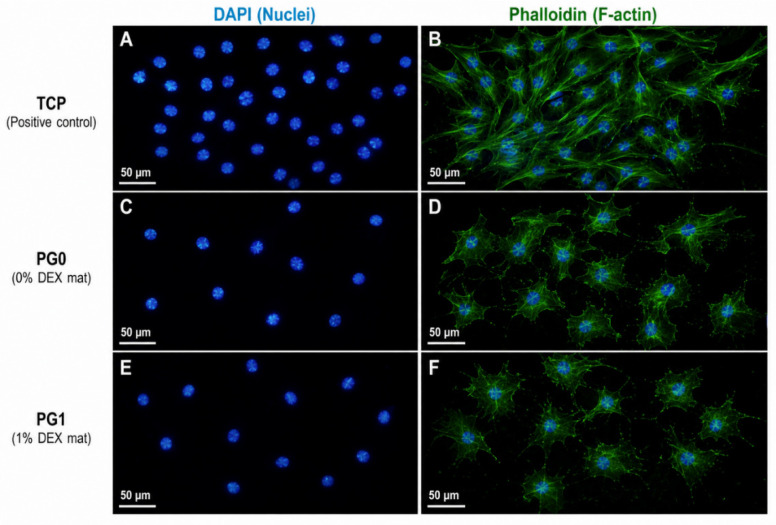
Cellular adhesion and cytoskeletal organisation on nanofibre mats. Fluorescence images of fibroblasts cultured for 24 h. Nuclei were stained with Hoechst 33342 (blue) and F-actin was stained with phalloidin (green). (**A**) Cells cultured on TCP (positive control) showing DAPI-stained nuclei; (**B**) F-actin organisation on TCP (positive control); (**C**) Cells cultured on PG0 (0% DEX mat) showing DAPI-stained nuclei; (**D**) F-actin organisation on PG0 (0% DEX mat); (**E**) Cells cultured on PG1 (1% DEX mat) showing DAPI-stained nuclei; (**F**) F-actin organisation on PG1 (1% DEX mat).

Cell adhesion and cytoskeletal organisation are not affected by the presence of 1 wt% DEX, which shows that the polymer matrix retains its biointeractive function after drug loading. This is very important for wound-healing application, where interaction of scaffold with cell plays a crucial role in tissue regeneration [30]. Cellular adhesion and cytoskeletal organisation on nanofibre mats are shown in Figure 6.

### 3.7. Structure–Transport Coupling in Drug Release

**Figure 7:**
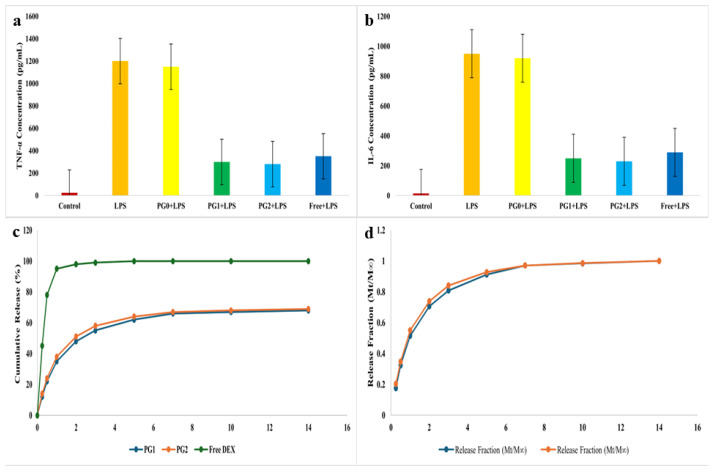
Anti-inflammatory response and drug release behaviour of DEX-loaded nanofibres. (**a**) TNF-α and (**b**) IL-6 concentrations in LPS-stimulated RAW 264.7 macrophages. (**c**) Cumulative release profiles of dexamethasone over time. (**d**) Normalised release kinetics (Mt/M∞).

The drug release profiles (Figure 7c,d) show a significant change in release characteristics with an increase in DEX loading. PG2 showed a very high initial burst release, while PG1 showed a relatively slow and controlled release pattern.

This variation is directly related to the morphological observations in Figure 3. The high density of crystals on the surface of PG2 provides a readily available reservoir of drug and results in a fast dissolution in aqueous environments. On the other hand, PG1 has a higher fraction of drug entrapped inside, which has to diffuse out of the polymer matrix.

The release mechanism can therefore be described as a two-component system:

- Surface-associated drug → burst release
- Matrix-embedded drug → diffusion-controlled release

As the drug loading changes, the relative contribution of these changes forms a direct structure–transport relationship. This is an important finding because it demonstrates that drug loading does not simply scale the amount of drug released but actually alters the release mechanism itself [31,32].

### 3.8. Macrophage Modulation and Biological Outcome

The anti-inflammatory response (Figure 7a,b) showed that the release of TNF-α and IL-6 in LPS-stimulated macrophages was significantly suppressed by both PG1 and PG2, with an inhibition of ~70–75%. This suggests that DEX is bioactive after electrospinning and can effectively modulate inflammatory signalling pathways.

Quantitative ELISA analysis demonstrated substantial reductions in both TNF-α and IL-6 production following treatment with DEX-loaded nanofibres. These cytokines are key mediators of chronic inflammation and delayed wound healing. The observed suppression confirms that dexamethasone remained biologically active following electrospinning and was released at therapeutically relevant concentrations capable of modulating macrophage inflammatory responses.

But a more nuanced picture emerges when read in light of the release and cytocompatibility data. PG2 has a burst release profile, which leads to rapid cytokine suppression but is associated with decreased cell viability. PG1, on the other hand, showed sustained drug release, similar anti-inflammatory efficacy and improved cytocompatibility.

This suggests that the biological performance depends on the drug dose as well as the release dynamics, emphasising the importance of controlling drug distribution in the fibre [20].

### 3.9. Degradation-Driven Structural Evolution

**Figure 8:**
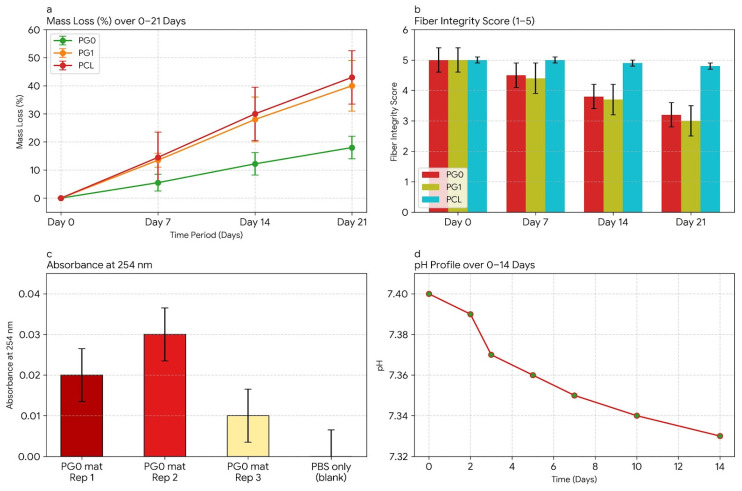
Degradation behaviour of electrospun nanofibre mats in PBS (pH 7.4) at 37 °C. (**a**) Mass loss vs. time, (**b**) fibre integrity score, (**c**) absorbance variation, and (**d**) pH change.

Degradation analysis (Figure 8a–d) revealed that there is a gradual mass loss and morphological alteration in 21 days. Gelatin promotes hydrolytic degradation and PCL provides structural stability in the initial stages.

SEM analysis showed a transition from intact fibres to swollen and fused structures, leading to fragmentation and increased porosity. Structural evolution facilitates water ingress and supports sustained drug diffusion later on.

The constant pH value during the degradation period indicates that the system does not produce harmful acidic byproducts and is therefore physiologically compatible.

### 3.10. Integrated Performance and Optimal Design Window

The combined results clearly and consistently establish a structure–function relationship:

- DEX loading → phase-separation and surface crystallisation
- Phase distribution → release mechanism (burst vs. diffusion)
- Release behaviour→ macrophage modulation.
- High loading → cytotoxicity + mechanical compromise

This chain of causality shows that high drug loading is not optimal. Instead, PG1 is a crucial intermediate regime where:

- Control of crystallisation on surfaces
- Controlled rather than excessive release
- Retention of cytocompatibility
- Mechanical integrity remains adequate

**Figure 9:**
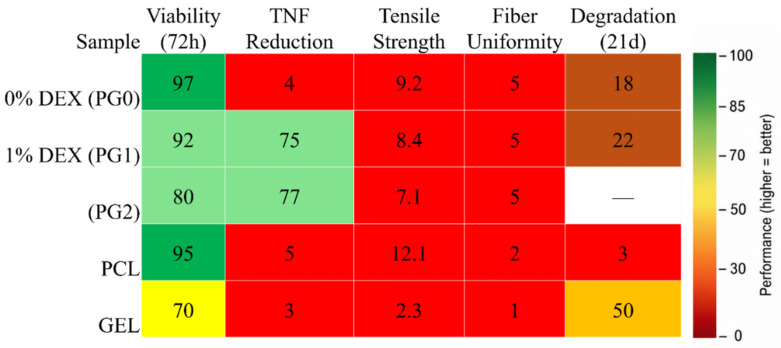
Heatmap summarising cytocompatibility, anti-inflammatory activity, mechanical properties, and degradation behaviour for PG0, PG1, and PG2.

Therefore, the system exhibits a dose-dependent window of performance, where the therapeutic efficacy is maximised without compromising the material or biological properties. Figure 9 demonstrate the heatmap summarising cytocompatibility, anti-inflammatory activity, mechanical properties, and degradation behaviour for PG0, PG1, and PG2.

## 4. Discussion

Several studies have reported on dexamethasone-loaded electrospun nanofibrous systems for localised anti-inflammatory therapy and wound-healing applications with focus on drug incorporation efficiency, sustained release behaviour and therapeutic efficacy [33,34,35]. However, the mechanistic role of the drug phase distribution within electrospun fibres is poorly understood. In the majority of the existing reports, drug loading is treated as a compositional parameter with an implicit assumption that with the increase in drug content, the therapeutic performance will be proportionally improved [35,36,37]. However, during electrospinning, rapid solvent evaporation can induce supersaturation-driven phase separation and crystallisation of low-molecular-weight therapeutics, fundamentally altering drug accessibility and transport pathways [38,39,40]. Heterogeneous drug distribution in electrospun systems is accepted, but its direct relation with the release kinetics, cytocompatibility and immunomodulatory response has rarely been systematically established.

To fill this gap, the present study shows that the efficacy of drug-loaded electrospun nanofibres is determined not only by the drug loading but also by the spatial distribution of drug in the fibre matrix, which dictates the release kinetics and subsequent biological response. Our results show that increasing dexamethasone loading fundamentally changes the phase behaviour, leading to a transition from relatively homogeneous incorporation to the formation of surface-localised crystalline domains. This structural evolution directly controls the transport behaviours and the subsequent biological outcomes and thus defines a clear structure–transport–bioactivity relationship in the electrospun system.

A key finding of this work is the finding of dose-dependent surface crystallisation as the dominant structural feature that controls drug transport. SEM analysis shows the evolution from a homogeneous polymer–drug system to a phase-separated system with crystalline domains localised on the surface by increasing DEX content. The observed behaviour is consistent with supersaturation-induced nucleation during rapid solvent evaporation, where limited drug solubility in the polymer matrix favours migration to energetically favourable interfaces. Similar phase separation phenomena have been reported in electrospun systems, but their implications for biological performance have rarely been systematically explored. This observation is in line with previous work by Rasti Boroojen et al., who observed weak polymer–drug interactions in PCL/gelatin/DEX systems but did not systematically explore dose-dependent surface crystallisation and its immunomodulatory consequences.

The correlation of the morphology to release kinetics demonstrates that the transport behaviour is directly controlled by this phase transition. At higher loading (PG2), surface drug dominates and leads to a burst release profile, while at intermediate loading (PG1), a mixture of surface-accessible and matrix-embedded drug provides a balanced release regime. This is a key difference as it shows not only the increased amount of release due to drug loading but also a change in the release mechanism from diffusion-controlled to surface-dominated transport.

Importantly, these differences in release behaviour directly influence the biological response. Both PG1 and PG2 demonstrate a significant suppression of pro-inflammatory cytokines, confirming the bioactivity of dexamethasone after the electrospinning. But the increased release rate of PG2 results in increased local concentrations of drug, which are associated with decreased fibroblast viability. PG1, in contrast, displayed high cytocompatibility and comparable anti-inflammatory efficacy. The results suggest that controlled release, rather than rapid release, can improve the ability to sustain therapeutic function without cytotoxic effects.

This result exemplifies the fundamental design principle that the maximisation of drug loading does not equal the optimisation of therapeutic performance. Instead, there is a dose-dependent window of performance balancing drug distribution, release kinetics and biological compatibility. The current system has an optimal regime matching PG1, which allows for adequate drug availability to modulate macrophages without excessive burst release and structural compromise. To our knowledge, such a non-monotonic performance window, where intermediate loading (1 wt%) outperforms higher loading (2 wt%) for the combined metrics of release kinetics, cytocompatibility, mechanical integrity, and anti-inflammatory efficacy, has not been previously demonstrated in electrospun dexamethasone delivery systems.

This interpretation is further supported by mechanical and degradation analyses. The increase in the drug content leads to microstructural heterogeneity, which reduces the mechanical integrity by disrupting the polymer chain interactions and creating stress concentration sites. On the other hand, the degradation behaviour is controlled by the PCL/gelatin matrix, with gelatin resulting in a controlled hydrolytic degradation without significant pH change. Together, these results suggest that structural and functional properties are closely related and that drug-induced phase behaviour needs to be considered with mechanical and degradation performance in material design.

This work gives a more complete framework than previously reported electrospun drug delivery systems by explicitly linking microstructure, transport, and biological function. The anti-inflammatory effects of DEX-loaded fibres have been reported in earlier studies; however, the effect of drug phase distribution on release behaviour and cytocompatibility has been largely ignored. The identification of surface crystallisation as a governing parameter is a step toward the mechanistically informed design of electrospun therapeutics beyond empirical formulation.

Comparison with previously reported electrospun PCL/gelatin systems indicates that the mechanical properties observed in the present study fall within the range generally reported for wound-healing scaffolds while maintaining high cytocompatibility. Unlike many previous studies that primarily correlate drug loading with therapeutic outcome, the present work demonstrates that drug concentration influences phase distribution and surface morphology, which subsequently affect release behaviour, mechanical performance, and biological response. These findings highlight the importance of considering structural evolution alongside drug concentration when designing electrospun therapeutic systems.

There are several limitations to acknowledge. 1. The evaluation is limited to in vitro, and in vivo wound-healing models are required to confirm the translational efficacy. Secondly, the long-term release beyond the study period was not investigated and this might be relevant for chronic wound applications. Finally, although dexamethasone possesses potent anti-inflammatory effects, combination strategies with antimicrobial or pro-regenerative agents may enhance the therapeutic efficacy. While SEM observations strongly suggest the formation of surface-associated crystalline domains at higher drug loading, definitive confirmation of crystallinity would benefit from complementary XRD and DSC analyses. Such investigations will be included in future studies to quantitatively evaluate crystal formation and drug phase transitions within the electrospun matrix.

Drug loading efficiency and encapsulation efficiency were not quantified using HPLC in the present work. Future studies will include validated HPLC-based analysis to accurately determine drug encapsulation efficiency and correlate loading characteristics with release behaviour and biological performance.

Future investigations should include antibacterial activity assessment, hemocompatibility testing, exudate absorption capacity, water vapour transmission rate, oxygen permeability, in vivo wound-healing evaluation, and long-term safety studies. These analyses will provide a more comprehensive assessment of clinical applicability as wound dressing materials.

Solution rheological properties, including viscosity, viscoelastic behaviour, and temperature-dependent flow characteristics, were not measured in this study. Future investigations should evaluate how dexamethasone incorporation influences solution rheology and how these parameters affect jet stability, phase separation, and final fibre morphology during electrospinning.

Despite these limitations, the results establish a clear structure–function relationship and provide design guidelines that are generally applicable to drug-loaded electrospun systems. The ability to control phase distribution of drug rather than just maximise drug content is a shift in design strategy that could be applied to other therapeutic molecules and polymer systems.

## 5. Conclusions

This study demonstrates that the performance of electrospun dexamethasone-loaded PCL/gelatin nanofibres is governed by dose-dependent surface crystallisation that affects drug release kinetics and subsequent biological response. Increasing drug loading leads to a transformation from homogeneous fibre structures to phase-separated systems with surface-located crystalline domains, thus changing from diffusion-controlled to burst-dominated release.

High drug loading, although enhancing initial drug availability, also induces cytotoxic effects and compromises mechanical integrity. In contrast, an intermediate loading (1 wt%) leads to a balanced system, combining a controlled release, high cytocompatibility and effective anti-inflammatory activity. This identifies a dose-dependent performance window in which therapeutic efficacy is maximised without compromising material or biological properties.

By establishing a direct link between drug phase behaviour, transport mechanisms, and macrophage modulation, this work provides a mechanistic framework for the rational design of electrospun drug delivery systems. The findings highlight that optimisation of drug distribution, rather than maximisation of drug content, is critical for achieving superior therapeutic performance, offering new design principles for advanced wound-healing materials.

While these in vitro results establish a strong mechanistic foundation, future in vivo wound-healing studies in appropriate animal models are necessary to confirm translational potential and therapeutic efficacy in a physiologically relevant wound microenvironment.

## Data Availability

The data presented in this study are available on request from the corresponding authors.
